# The Scaled Hirshfeld Partitioning: Mathematical Development and Information-Theoretic Foundation

**DOI:** 10.3390/e28030362

**Published:** 2026-03-23

**Authors:** Farnaz Heidar-Zadeh

**Affiliations:** Department of Chemistry, Queen’s University, 90 Bader Lane, Kingston, ON K7L-3N6, Canada; farnaz.heidarzadeh@queensu.ca

**Keywords:** atoms in molecules, atomic charges, Hirshfeld partitioning methods, scaled Hirshfeld, additive variational Hirshfeld, information-theoretic atoms, condensed reactivity descriptors

## Abstract

Atomic charges play a central role in the analysis of molecular electronic structure and are widely used in the development of computational models. We introduce a simple and computationally efficient extension of Hirshfeld’s 1977 stockholder partitioning method, called scaled Hirshfeld, in which neutral proatom densities are scaled to construct a promolecular density better adapted to the molecular electron density. We present a fixed-point iterative algorithm to compute the proatom scaling coefficients and show that this formulation is equivalent to the information-theoretic additive variational Hirshfeld method with a minimal basis. This equivalence establishes a rigorous mathematical foundation for the scaled Hirshfeld method and ensures size consistency as well as the existence of a unique solution. Numerical results demonstrate that the proposed approach yields charges larger than those obtained with the original Hirshfeld method, while retaining computational efficiency and providing an improved description of molecular dipole moments and electrostatic potentials.

## 1. Introduction

Although atoms in molecules are not rigorously defined within the framework of quantum chemistry, they remain useful and widely used constructs for both the quantitative and qualitative analysis of molecular bonding, interactions, and reactivity [1,2,3,4,5,6,7,8,9,10,11,12,13,14,15]. As a result, numerous partitioning schemes have been developed to define atomic electron densities and associated atomic properties. These approaches go beyond traditional population analysis methods, which typically aim only to assign atomic charges and are often basis-set-dependent, by providing spatially resolved atomic densities from which a wide range of atomic properties, such as atomic moments, polarizabilities, and dispersion coefficients, can be computed and employed in force field development and modeling intermolecular interactions [16,17,18,19,20,21,22,23,24,25].

Density-based partitioning schemes decompose the molecular electron density $\rho_{\mathrm{mol}}\left(r\right)$ into atom-centered contributions $\left\{\rho_{A}\left(r\right)\right\}$ such that their sum exactly reproduces the molecular density at every point in space.

(1) $$\rho_{\mathrm{mol}}\left(r\right)=\sum_{A}\rho_{A}\left(r\right).$$

This equation can equivalently be expressed in terms of atomic weight functions $\left\{w_{A}\left(r\right)\right\}$, which specify the fraction of the molecular electron density assigned to each atom at a given point in space.

(2) $$\rho_{A}\left(r\right)=w_{A}\left(r\right)\;\rho_{\mathrm{mol}}\left(r\right)\;s.t.\;\forall r:w_{A}\left(r\right)\geq 0\;\mathrm{and}\;\sum_{A}w_{A}\left(r\right)=1$$

As a result, atomic densities are fully determined by the choice of atomic weight functions, which can be broadly classified into two categories. Binary schemes assign each point in space exclusively to a single atom, resulting in weights $w_{A}\left(r\right)\in \left\{0,1\right\}$ [1,26,27,28,29]. In contrast, fuzzy schemes allow overlapping atomic regions with continuous weights $w_{A}\left(r\right)\in \left[0,1\right]$ [29,30,31]. Among fuzzy partitioning methods, the family of Hirshfeld schemes employs a stockholder approach, in which atomic weight functions are defined in terms of atom-centered proatom densities $\left\{\rho_{A}^{0}\left(r\right)\right\}$,

(3) $$w_{A}\left(r\right)=\frac{\rho_{A}^{0}\left(r\right)}{\rho_{\mathrm{mol}}^{0}\left(r\right)}=\frac{\rho_{A}^{0}\left(r\right)}{\sum_{B}\rho_{B}^{0}\left(r\right)}$$

where the denominator represents the promolecular density constructed as the sum of the proatom densities [32]. Originally proposed heuristically by Hirshfeld in 1977, the atomic weight function in Equation (3) was later shown to possess a rigorous information-theoretic foundation [30,33]. In particular, the resulting atomic densities are those that minimize an *f*-divergence from the reference proatom densities, subject to the constraint that their sum reproduces the molecular electron density, as expressed in Equation (1) [33]. For a fixed set of proatoms, the optimal atomic densities $\rho_{A}\left(r\right)$ are obtained by solving

(4) $$\left\{\rho_{A}\left(r\right)\right\}=\arg\underset{\left\{\rho_{A}\left(r\right)|\rho_{\mathrm{mol}}\left(r\right)=\sum_{A}\rho_{A}\left(r\right)\right\}}{\min_{︸}}\sum_{A}\int \rho_{A}\left(r\right)f\left(\frac{\rho_{A}^{0}\left(r\right)}{\rho_{A}\left(r\right)}\right)dr$$

where *f* is any convex function satisfying $f\left(1\right)=f^{\prime }\left(1\right)=0$. In addition, it was demonstrated that the family of *f*-divergences is both necessary and sufficient for deriving Hirshfeld atomic densities [33]. This information-theoretic perspective demonstrates the pervasiveness of the Hirshfeld partitioning scheme, thereby unifying earlier formulations based on specific choices such as the Kullback–Leibler divergence (f(x)=−lnx) and the squared Hellinger–Bhattacharyya distance ($f\left(x\right)=\frac{1}{2}{\left(\sqrt{x}-1\right)}^{2}$) [34,35,36,37,38,39,40].

However, the choice of proatom densities remains a central challenge. In particular, the use of fixed neutral proatoms in the original Hirshfeld method is known to systematically underestimate atomic charges and to perform poorly for modeling molecular interactions [41]. To resolve this, numerous Hirshfeld variants have been developed that identify system-specific proatoms, leading to improved atomic densities and properties [42,43,44,45,46,47,48,49,50,51,52,53,54]. Some of these methods result in atomic charges that, by construction, reproduce the molecular dipole moment [55,56,57,58]. In parallel, reference-free partitioning approaches have also been developed that bypass proatoms altogether [59]. Leveraging the established information-theoretic framework in Equation (4), we introduced a variational approach for optimizing proatom densities, such that the molecule and promolecule have the same number of electrons:

(5) $$\left\{\rho_{A}^{0}\left(r\right)\right\}=\arg\underset{\left\{\rho_{A}^{0}\left(r\right)|N_{\mathrm{mol}}=\int \rho_{\mathrm{mol}}^{0}\left(r\right)\;dr\right\}}{\min_{︸}}\int \rho_{\mathrm{mol}}\left(r\right)f\left(\frac{\rho_{\mathrm{mol}}^{0}\left(r\right)}{\rho_{\mathrm{mol}}\left(r\right)}\right)\;dr$$

where $N_{\mathrm{mol}}=\int \rho_{\mathrm{mol}}\left(r\right)\;dr$ denotes the total number of electrons in the molecular density. This led to the development of the minimal-basis iterative stockholder (MBIS) [46] and additive variational Hirshfeld (AVH) [49] methods. These approaches use different proatom models in Equation (5), but both variationally optimize (non)linear proatom parameters by minimizing the extended Kullback–Leibler divergence (f(x)=−ln(x)+x−1) between the molecular and promolecular electron densities. The extended Kullback–Leibler divergence is specifically used because, by default, the proatom (promolecule) and atom (molecule) will have the same number of electrons, eliminating the need for an additional constraint in the optimization process.

In the present work, we introduce a simple and computationally efficient extension of the original Hirshfeld partitioning, called scaled Hirshfeld, in which neutral proatom densities are scaled to construct a promolecule better adapted to the molecular electron density. In this approach, promolecular density is expressed as a linear combination of neutral reference proatoms, and an iterative algorithm is proposed to self-consistently optimize the scaling coefficients for a given system. We further provide a variational formulation of the scaled Hirshfeld method based on information theory and demonstrate that it is equivalent to the additive variational Hirshfeld method with a minimal basis. This connection strengthens the mathematical foundation of scaled Hirshfeld partitioning. Finally, we present numerical results showing that this simple modification of proatoms leads to significant improvements over standard Hirshfeld charges.

## 2. Development of Scaled Hirshfeld

In this section, we introduce a fixed-point iteration formulation of the scaled Hirshfeld (SH) partitioning scheme to determine optimal proatoms through the scaling of reference densities. In this framework, the promolecular density $\rho_{\mathrm{mol}}^{0}$ is constructed as a linear combination of spherically averaged neutral isolated reference densities $\left\{\rho_{A}^{0}\left(r\right)\right\}$; that is,

(6) $$\rho_{\mathrm{mol}}^{0}\left(r;\left\{c_{A}\right\}\right)=\sum_{A}\rho_{A}^{0}\left(r;c_{A}\right)=\sum_{A}c_{A}\;\rho_{A}^{0}\left(r\right),\;c_{A}>0,$$

where $\rho_{A}^{0}\left(r;c_{A}\right)=c_{A}\;\rho_{A}^{0}\left(r\right)$ denotes the proatom density of atom *A*, obtained by scaling the corresponding reference density by the positive coefficient $c_{A}$. The total promolecular electron population is given by $N_{\mathrm{mol}}^{0}=\sum_{A}N_{A}^{0}$, where each proatom population $N_{A}^{0}$ is obtained by scaling the reference atomic population $n_{A}^{0}$. Explicitly,

(7) $$N_{A}^{0}=\int \rho_{A}^{0}\left(r;c_{A}\right)\;dr=c_{A}\int \rho_{A}^{0}\left(r\right)\;dr=c_{A}\;n_{A}^{0}.$$

Given the promolecular density defined in Equation (6), the atomic weight function of the SH partitioning is defined as

(8) $$w_{A}\left(r;\left\{c_{A}\right\}\right)=\frac{\rho_{A}^{0}\left(r;c_{A}\right)}{\rho_{\mathrm{mol}}^{0}\left(r;\left\{c_{A}\right\}\right)}=\frac{c_{A}\;\rho_{A}^{0}\left(r\right)}{\rho_{\mathrm{mol}}^{0}\left(r;\left\{c_{A}\right\}\right)}\;$$

Varying the coefficients $\left\{c_{A}\right\}$ therefore rescales the neutral reference populations, which in turn modifies both the proatom densities and the resulting promolecular density. To determine the optimal set of coefficients for a given molecule, we adapt a fixed-point iteration scheme analogous to the Iterative Hirshfeld (HI) method [42,60]. In this approach, the proatoms are optimized through a self-consistent approach by stipulating that the proatom populations match the atomic populations obtained by partitioning the molecular electron density. Specifically, at the (k+1)-th iteration, the atomic populations are obtained by integrating the corresponding atomic densities constructed from the proatom densities that match the atomic populations of the (k)-th iteration:

(9) $$N_{A}^{\left(k+1\right)}=\int \rho_{\mathrm{mol}}\left(r\right)\;w_{A}^{\left(k\right)}\left(r\right)\;dr,\;\mathrm{where}\;w_{A}^{\left(k\right)}\left(r\right)=\frac{c_{A}^{\left(k\right)}\rho_{A}^{0}\left(r\right)}{\sum_{B}c_{B}^{\left(k\right)}\rho_{B}^{0}\left(r\right)}.$$

Here, the integrand represents the atomic electron density of atom *A* at the (k+1)-th iteration, i.e., $\rho_{\mathrm{mol}}\;\left(r\right)\;w_{A}^{\left(k\right)}\;\left(r\right)=\rho_{A}^{\left(k+1\right)}\;\left(r\right)$. Note that in Equation (9), for simplicity, we write $w_{A}\left(r\right)$ instead of $w_{A}\left(r;\left\{c_{A}\right\}\right)$, thereby omitting the explicit dependence on the set of scaling coefficients $\left\{c_{A}\right\}$. The scaling coefficients are then updated by enforcing equality between the proatom and atomic populations.

(10) $$c_{A}^{\left(k\right)}=\frac{N_{A}^{\left(k\right)}}{n_{A}^{0}}.$$

This iterative procedure is repeated until the convergence of the scaling coefficients, i.e., $c_{A}^{\left(k+1\right)}\approx c_{A}^{\left(k\right)}$ for all *A*, or, equivalently, until the convergence of the atomic populations, i.e., $N_{A}^{\left(k+1\right)}\approx N_{A}^{\left(k\right)}$ for all *A*. This results in a self-consistent set of scaled proatom densities and the corresponding promolecular density of the SH approach. Algorithm 1 presents pseudocode for SH, clearly summarizing the steps involved. As with any iterative scheme, an initial guess and user-defined convergence threshold are required; here, we use the original Hirshfeld partitioning as the starting point, i.e., $c_{A}=1$ for all atoms. In the following section, we demonstrate that this fixed-point iteration corresponds to a convex optimization problem, ensuring the existence of a unique solution $\left\{c_{A}\right\}$ that is independent of the initial guess.
**Algorithm 1:** Scaled Hirshfeld (SH) Fixed-Point Iteration**Inputs:** Molecular density $\rho_{\mathrm{mol}}\left(r\right)$; neutral reference proatom densities $\left\{\rho_{A}^{0}\left(r\right)\right\}$; reference  proatom populations $n_{A}^{0}=\int \rho_{A}^{0}\left(r\right)\;dr$; convergence threshold ε; maximum iterations $K_{\max}$.  **Outputs:** Scaling coefficients $\left\{c_{A}\right\}$; atomic weights $\left\{w_{A}\left(r\right)\right\}$; atomic populations $\left\{N_{A}\right\}$.  **Initialization:** Set $c_{A}^{\left(0\right)}\leftarrow 1$ for all atoms *A*; Set k←0.  While $k<K_{\max}$:       1. Construct promolecular density:$$\rho_{\mathrm{mol}}^{0,(k)}\left(r\right)=\sum_{B}c_{B}^{\left(k\right)}\rho_{B}^{0}\left(r\right)$$       2. Compute atomic weight functions:$$w_{A}^{\left(k\right)}\left(r\right)=\frac{c_{A}^{\left(k\right)}\rho_{A}^{0}\left(r\right)}{\rho_{\mathrm{mol}}^{0,(k)}\left(r\right)}$$       3. Update atomic populations:$$N_{A}^{\left(k+1\right)}=\int \rho_{\mathrm{mol}}\left(r\right)\;w_{A}^{\left(k\right)}\left(r\right)\;dr$$       4. Update scaling coefficients:$$c_{A}^{\left(k+1\right)}=\frac{N_{A}^{\left(k+1\right)}}{n_{A}^{0}}$$       5. If $\max_{A}\left|c_{A}^{\left(k+1\right)}-c_{A}^{\left(k\right)}\right|<\varepsilon$ for all atoms, stop the iteration. Otherwise, set k←k+1.  Compute the final atomic densities:$$\rho_{A}\left(r\right)=\rho_{\mathrm{mol}}\left(r\right)\;w_{A}^{\left(k\right)}\left(r\right)$$

## 3. Results and Discussions

We first demonstrate that the SH algorithm introduced in Section 2 is the fixed-point formulation of the constrained minimization of an extended Kullback–Leibler divergence between the promolecular and molecular electron densities. This result establishes that the proposed iterative SH algorithm, despite its simplicity and ease of implementation, is formally equivalent to the additive variational Hirshfeld method with a minimal basis (AVH-M) [49]. We then present numerical results showing that the atomic charges obtained from the SH method systematically improve original Hirshfeld charges and closely resemble Charge Model 5 (CM5) charges [61], which empirically refine Hirshfeld charges to better reproduce molecular observables such as dipole moments.

### 3.1. Variational Formulation of Scaled Hirshfeld

The promolecular model defined in Equation (6) is identical to that employed in AVH-M [49]. Within the additive variational Hirshfeld (AVH) framework, AVH-M employs the smallest possible basis expansion, making it computationally efficient, by representing each element with its spherically averaged ground-state neutral proatom density as the basis function. The corresponding basis coefficients scale these proatom densities such that the resulting promolecular density provides the best approximation to the molecular electron density. Mathematically, this procedure corresponds to minimizing the extended Kullback–Leibler (ext-KL) divergence between the molecular and promolecular electron densities, subject to the constraint that both densities integrate to the same total number of electrons. Using the promolecular density defined in Equation (6) and the proatom populations given in Equation (7), this constrained optimization problem can be written using the $f_{\text{ext-KL}}\left(x\right)=-\ln\left(x\right)+x-1$ in Equation (5); that is,

(11) $$\begin{array}{cc} \{c_{A}\}\; & =\arg\min_{\left\{\left\{c_{A}>0\right\}\;|\;N_{\mathrm{mol}}=N_{\mathrm{mol}}^{0}=\sum_{A}c_{A}n_{A}^{0}\right\}}\\ \; & \;\int \left[\rho_{\mathrm{mol}}\left(r\right)\ln\;\left(\frac{\rho_{\mathrm{mol}}\left(r\right)}{\rho_{\mathrm{mol}}^{0}\left(r;\left\{c_{A}\right\}\right)}\right)+\rho_{\mathrm{mol}}^{0}\left(r;\left\{c_{A}\right\}\right)-\rho_{\mathrm{mol}}\left(r\right)\right]dr. \end{array}$$

where $N_{\mathrm{mol}}=\int \rho_{\mathrm{mol}}\left(r\right)\;dr$ denotes the total number of electrons in the molecule. Using the optimal $\left\{c_{A}\right\}$, the AVH-M atomic weight function is calculated using Equation (8) similarly to the scaled Hirshfeld approach.

We now prove that the stationary point of Equation (11) satisfies the fixed-point iteration defined in Equation (9). To determine the optimal coefficients, we construct the Lagrangian associated with the constrained minimization problem in Equation (11). Dropping terms that are independent of $\left\{c_{A}\right\}$ and using the Lagrange multiplier λ, the Lagrangian takes the form

(12) $$L\left(\left\{c_{A}\right\},\lambda \right)=\int \left[\rho_{\mathrm{mol}}\left(r\right)\ln\;\left(\frac{\rho_{\mathrm{mol}}\left(r\right)}{\rho_{\mathrm{mol}}^{0}\left(r;\left\{c_{A}\right\}\right)}\right)+\rho_{\mathrm{mol}}^{0}\left(r;\left\{c_{A}\right\}\right)\right]dr+\lambda \;\left(\sum_{A}c_{A}n_{A}^{0}-N_{\mathrm{mol}}\right).$$

where $\left\{n_{A}^{0}\right\}$ is the reference atomic populations defined in Equation (7). The stationary conditions follow from setting the derivatives of L with respect to λ and $c_{A}$ to zero. Differentiating with respect to λ gives the normalization constraint:

(13) $$\frac{\partial L}{\partial \lambda }=0=\sum_{A}c_{A}n_{A}^{0}-N_{\mathrm{mol}}.$$

Similarly, differentiation with respect to coefficient $c_{A}$ gives

(14) $$\forall A:\;\frac{\partial L}{\partial c_{A}}=0=\int \left[-\rho_{\mathrm{mol}}\left(r\right)\;\left(\frac{\rho_{A}^{0}\left(r\right)}{\rho_{\mathrm{mol}}^{0}\left(r;\left\{c_{A}\right\}\right)}\right)+\rho_{A}^{0}\left(r\right)\right]dr+\lambda \;n_{A}^{0}$$

which, using $\int \rho_{A}^{0}\left(r\right)\;dr=n_{A}^{0}$, simplifies to

(15) $$\;\int \rho_{\mathrm{mol}}\left(r\right)\;\frac{\rho_{A}^{0}\left(r\right)}{\rho_{\mathrm{mol}}^{0}\left(r;\left\{c_{A}\right\}\right)}\;dr=\left(1+\lambda \right)\;n_{A}^{0}.$$

Incorporating the atomic weight functions defined in Equation (8), this condition can be rewritten as

(16) $$\int \rho_{\mathrm{mol}}\left(r\right)\;\frac{w_{A}\left(r;\left\{c_{A}\right\}\right)}{c_{A}}\;dr=\left(1+\lambda \right)\;n_{A}^{0},$$

or equivalently

(17) $$c_{A}=\frac{1}{\left(1+\lambda \right)\;n_{A}^{0}}\int \rho_{\mathrm{mol}}\left(r\right)\;w_{A}\left(r;\left\{c_{A}\right\}\right)\;dr.$$

Multiplying both sides by $n_{A}^{0}$ and summing over all atoms, and using the partition-of-unity property $\sum_{A}w_{A}\left(r;\left\{c_{A}\right\}\right)=1$, yields

(18) $$\sum_{A}c_{A}n_{A}^{0}=\frac{1}{1+\lambda }\int \rho_{\mathrm{mol}}\left(r\right)\;dr=\frac{N_{\mathrm{mol}}}{1+\lambda }$$

Together with the normalization constraint in Equation (13), this implies λ=0, which leads to the fixed-point condition:

(19) $$c_{A}=\frac{1}{n_{A}^{0}}\int \rho_{\mathrm{mol}}\left(r\right)\;w_{A}\left(r;\left\{c_{A}\right\}\right)\;dr.$$

This equation defines a self-consistency condition for the scaling coefficients $\left\{c_{A}\right\}$; that is, evaluating the atomic weights using coefficients from the previous iteration yields the fixed-point update rule in Equations (9) and (10). This establishes that the SH algorithm is the fixed-point iteration formulation of the variational optimization in AVH-M.

### 3.2. Numerical Results

We used a dataset of organic molecules to assess the performance of the scaled Hirshfeld (SH) method against the original Hirshfeld (H) [32] and the additive variational Hirshfeld method with a bound basis (AVH-B) [49], which employs a larger set of reference densities to construct the promolecule. Note that, as demonstrated in Section 3.1, SH charges are identical to those obtained from the AVH-M method. In addition, we considered the Hu–Lu–Yang atomic charges (HLYGAt) [62] and Charge Model 5 (CM5) [61], which refine Hirshfeld charges empirically to reproduce charge-dependent molecular observables such as dipole moments. Although only a single level of theory is used in this study, previous work demonstrating the insensitivity of AVH-M to the level of theory and basis set indicates the computational robustness of SH [49]. This behavior is expected because, similar to the original Hirshfeld method, SH relies exclusively on neutral proatom densities, which exhibit very little sensitivity to the level of theory.

**Figure 1:**
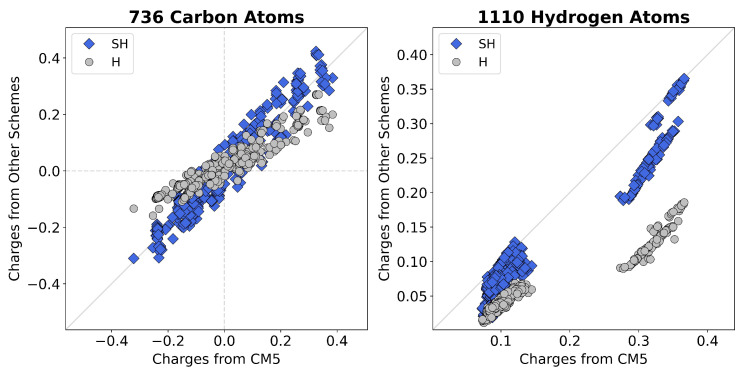
Comparison of atomic charges for carbon ((**left**), 736 atoms) and hydrogen ((**right**), 1110 atoms) across 168 organic molecules obtained using different charge schemes. Charges from the scaled Hirshfeld method (SH, blue diamonds) and the original Hirshfeld method (H, gray circles) are plotted against the corresponding Charge Model 5 (CM5) reference values. The diagonal line denotes perfect agreement between methods.

Table 1 assesses the pairwise agreement between charges from different schemes using correlation coefficients and linear-fit slopes. This reveals a significant correlation between SH and H ($R^{2}=0.922$) and SH and CM5 ($R^{2}=0.912$). However, the slope of H versus SH (0.555) indicates that SH charges are systematically larger. Conversely, the slope of SH versus CM5 (0.801) indicates that SH charges tend to be slightly smaller than CM5 charges. To further demonstrate this, Figure 1 plots the Hirshfeld (H) and scaled Hirshfeld (SH) charges against CM5 charges for carbon and hydrogen atoms. This comparison more clearly illustrates how these two refinements of the original Hirshfeld model relate to one another and highlights the utility of scaling neutral proatoms in improving charges.

To assess the performance of SH charges in reproducing molecular electrostatics, we evaluate two quantities for each molecule: (a) the norm of the error in the point-charge approximation to the molecular dipole moment [63,64] and (b) the mean absolute error (MAE) of the point-charge approximation to the electrostatic potential evaluated on the molecular van der Waals surface [49,65,66]. These quantities are defined as

(20) $$\begin{array}{cc} |\mu_{\mathrm{ref}}-\mu_{\mathrm{approx}}|\; & =\left|\mu -\sum_{A}q_{A}R_{A}\right|, \end{array}$$

(21) $$\begin{array}{cc} \sum_{k=1}^{K_{m}}\left|V_{\mathrm{ref}}\left(r_{k}\right)-V_{\mathrm{approx}}^{\left(0\right)}\left(r_{k}\right)\right|\; & =\sum_{k=1}^{K_{m}}\left|V_{\mathrm{ref}}\left(r_{k}\right)-\sum_{A}\frac{q_{A}}{\left|r_{k}-R_{A}\right|}\right|. \end{array}$$

Here, $\mu_{\mathrm{ref}}$ denotes the reference molecular dipole moment, $q_{A}$ and $R_{A}$ are the atomic charge and Cartesian coordinates of atom *A*, respectively, and $V_{\mathrm{ref}}\left(r_{k}\right)$ is the reference electrostatic potential (ESP) evaluated at point $r_{k}$ sampled on the 2.5-scaled van der Waals surface of each molecule. The quantity $V_{\mathrm{approx}}^{\left(0\right)}\left(r_{k}\right)$ corresponds to the electrostatic potential obtained from the point-charge approximation.

**Figure 2:**
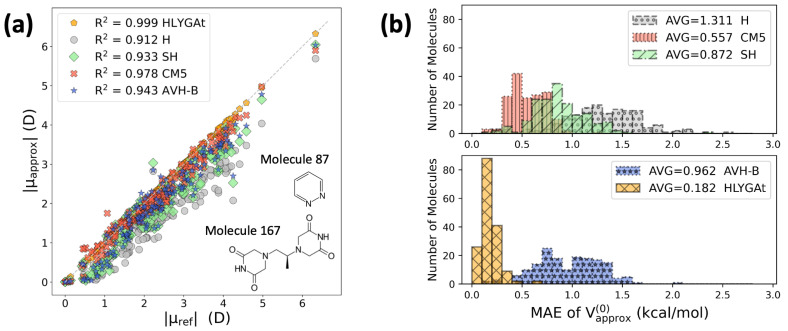
(**a**) Parity plot comparing the norm of the point-charge approximation to the molecular dipole moment (in Debye, D) with the norm of the reference values for 168 organic molecules; the legend reports the correlation coefficient for each charge scheme. (**b**) Distribution of the mean absolute error (MAE) of the point-charge approximation to the electrostatic potential for 168 organic molecules, with the legend reporting the average MAE for each scheme, consistent with the values in Table 2.

Table 2 summarizes the accuracy with which the different charge models reproduce molecular dipole moments and electrostatic potentials. As expected, the HLYGAt charges produce the lowest errors. The results clearly show that the SH method provides a significant improvement over the original Hirshfeld (H) scheme, with performance comparable to that of AVH-B, despite the latter employing a more flexible promolecular model. Moreover, SH errors are closer to those of CM5 than to H, consistent with the resemblance of their charges shown in Figure 1, reflecting the benefit of refining the neutral proatoms in the original Hirshfeld method to better represent the molecular electron density. To further support this analysis, a closer examination of errors associated with these point-charge approximations is provided in Figure 2. The maximum dipole errors (in Debye) are 0.151 (HLYGAt), 2.170 (H), 1.726 (SH), 0.761 (CM5), and 1.546 (AVH-B). The maximum electrostatic potential (ESP) errors (in kcal/mol) are 0.655 (HLYGAt), 2.89 (H), 1.23 (CM5), 2.33 (SH), and 2.01 (AVH-B). For both properties, the largest errors for H, SH, and AVH-B occur for molecule 87 (pyridazine), whereas the maximum error for CM5 is observed for molecule 167 (dexrazoxane), as shown in Figure 2.

#### Computational Details

The geometries for the 168 molecules in the organic dataset were taken from an earlier study [49], followed by a single-point gas-phase calculation at ωB97X-D/def2-TZVPD [67,68] level of theory with Gaussian16 (version C.01) [69] to obtain the wave function as well as the CM5 and HLYGAt charges. Atomic charges were then computed with the free and open source software packages from QC-Devs [70,71,72], including IOData (v1.0.1) [73], Grid (v0.0.9) [74], GBasis (v0.1.0) [75,76], and (our in-house and public) DensPart libraries (https://github.com/theochem/denspart, accessed on 8 March 2026); proatom densities were calculated at the same level of theory as the molecule. For the Hirshfeld-based partitioning schemes, a pruned Becke–Lebedev molecular grid called insane from the Grid [74] package was used, resulting in electron density integration errors below $5\times 10^{-4}$ a.u. for all molecules. The molecular electron density $\rho_{\mathrm{mol}}\left(r\right)$ on the 2.5-scaled van der Waals surface is below $5\times 10^{-7}$ a.u. for all molecules in the dataset, so the surface points are far from the molecular charge distribution.

## 4. Conclusions

We presented a simple modification to the promolecular density used in Hirshfeld’s 1977 scheme that leads to a clear improvement in the performance of atomic charges, particularly in their ability to approximate molecular permanent electrostatics. By allowing neutral proatom densities to be scaled in a self-consistent manner, the scaled Hirshfeld (SH) approach produces charges that are systematically larger than those obtained from the original Hirshfeld partitioning, while retaining its conceptual simplicity and computational efficiency. From this perspective, scaled Hirshfeld illustrates how much improvement can be achieved by refining only the populations of neutral proatoms, before introducing larger basis expansions or empirical corrections. This makes SH an attractive choice for applications that require improved electronic descriptions at minimal additional cost, including force field development and electrostatic potential modeling.

The variational formulation established here further demonstrates that the SH method is size-consistent and has a unique solution, revealing its equivalence to the additive variational Hirshfeld method with a minimal basis. This correspondence indicates that the improvements observed with the SH method arise from an underlying variational principle, ensuring that the resulting promolecular density is optimally adapted to the molecular electron density. In this sense, SH can be regarded as the simplest representative of a broader class of variationally optimal Hirshfeld partitioning schemes, providing a conceptual link between heuristic refinements of CM5 and fully variational approaches. More broadly, these findings highlight the importance of the information-theoretic foundation of Hirshfeld-based methods, particularly in guiding the construction of optimal promolecular densities.

## Figures and Tables

**Table 1:** Pairwise comparison of 2186 atomic charges calculated using different schemes for 168 organic molecules. Lower triangular elements report the squared Pearson correlation coefficient ($R^{2}$); upper triangular elements report the slopes of linear fits, where the row corresponds to the *y*-axis and the column to the *x*-axis.

Schemes	H	SH	CM5	AVH-B	HLYGAt
H	–	0.555	0.438	0.273	0.227
SH	0.922	–	0.801	0.421	0.414
CM5	0.817	0.912	–	0.475	0.473
AVH-B	0.528	0.420	0.375	–	0.446
HLYGAt	0.693	0.773	0.709	0.378	–

**Table 2:** Average errors, computed over 168 organic molecules, for point-charge approximations of molecular dipole moments in Equation (20) and electrostatic potentials (ESPs) in Equation (21) using different atomic charge models. Dipole errors are given in Debye (D) and ESP errors in kcal/mol.

Property	H	SH	CM5	AVH-B	HLYGAt
Dipole Norm (D)	0.667	0.407	0.179	0.354	0.019
ESP (kcal/mol)	1.311	0.872	0.557	0.962	0.182

## Data Availability

The atomic coordinates of the 168 organic molecules studied here, along with the corresponding atomic charges computed at the ωB97X-D/def2-TZVPD level, are provided as an extended XYZ file. For each molecule, the file reports the atomic number or symbol, Cartesian coordinates (X, Y, Z), followed by Hirshfeld (H), scaled Hirshfeld (SH), Charge Model 5 (CM5), additive variational Hirshfeld with bound proatoms (AVH-B), and Hu–Lu–Yang (HLYGAt) atomic charges. The dataset and instructions for loading and using it are available at https://github.com/qtchem/publications/tree/main/2026_entropy_scaled_hirshfeld (accessed on 8 March 2026).
